# Noninvasive quantification of granzyme B in cardiac allograft rejection using targeted ultrasound imaging

**DOI:** 10.3389/fimmu.2023.1164183

**Published:** 2023-06-26

**Authors:** Yunjie Jin, Peng Gao, Lifei Liang, Yuhang Wang, Jiawei Li, Jiyan Wang, Jiangang Hou, Cheng Yang, Xiaolin Wang

**Affiliations:** ^1^ Shanghai Institute of Medical Imaging, Shanghai, China; ^2^ Department of Ultrasound, Zhongshan Hospital, Fudan University, Shanghai, China; ^3^ Department of Urology, Huashan Hospital, Fudan University, Shanghai, China; ^4^ Department of Urology, Zhongshan Hospital, Fudan University, Shanghai, China; ^5^ Shanghai Key Laboratory of Organ Transplantation, Shanghai, China; ^6^ Shanghai Medical College, Fudan University, Shanghai, China; ^7^ Zhangjiang Institute of Fudan University, Shanghai, China

**Keywords:** cardiac transplantation, ultrasound imaging, rejection, contrast enhanced ultrasound (CEUS), granzyme B (GzmB)

## Abstract

**Objective:**

Endomyocardial biopsy is the gold standard method for the diagnosis of cardiac allograft rejection. However, it causes damage to the heart. In this study, we developed a noninvasive method for quantification of granzyme B (GzB) *in vivo* by targeted ultrasound imaging, which detects and provides quantitative information for specific molecules, for acute rejection assessment in a murine cardiac transplantation model.

**Methods:**

Microbubbles bearing anti-GzB antibodies (MB_Gzb_) or isotype antibodies (MBcon) were prepared. Hearts were transplanted from C57BL/6J (allogeneic) or C3H (syngeneic) donors to C3H recipients. Target ultrasound imaging was performed on Days 2 and 5 post-transplantations. A pathologic assessment was performed. The expression of granzyme B and IL-6 in the heart was detected by Western blotting.

**Results:**

After MB injection, we observed and collected data at 3 and 6 min before and after the flash pulse. Quantitative analysis revealed that the reduction in peak intensity was significantly higher in the allogeneic MB_Gzb_ group than in the allogeneic MB_con_ group and the isogeneic MB_con_ group at PODs 2 and 5. In the allogeneic groups, granzyme B and IL-6 expression levels were higher than those in the isogeneic group. In addition, more CD8 T cells and neutrophils were observed in the allogeneic groups.

**Conclusion:**

Ultrasound molecular imaging of granzyme B can be used as a noninvasive method for acute rejection detection after cardiac transplantation.

## 1 Introduction

Organ transplantation is a milestone in the medical history of humans that has saved millions of lives. Although novel immunosuppressive agents have been discovered and applied, the long-term survival of allografts is still not satisfactory. The major obstacle is rejection, including acute and chronic rejection. Therefore, it is quite beneficial to diagnose rejection in patients as early as possible. In the clinic, allograft biopsy and pathological examination are the gold standards for determining allograft immune status. For instance, in heart transplantation, physicians perform an endomyocardial biopsy, which helps in therapy decision-making. However, this procedure is invasive and has unavoidable perforation, arrhythmia, vessel damage, tricuspid regurgitation, and anesthetic-associated complications (1). Therefore, identifying noninvasive technologies for allograft immune status diagnosis is worthwhile.

Contrast-enhanced ultrasound (CEUS) imaging is a tolerable and noninvasive examination. The most frequently used ultrasound contrast agent is microbubbles (MBs), which are stabilized by a shell layer formed of lipids, proteins, or polymers (2). Furthermore, by labeling microbubbles with targeting materials, specific disease markers can be detected and quantified. Various intravascular targets have been proposed for microbubble targeting, such as integrins, selectins, and cell adhesion molecules (2, 3). Quantification can be performed *in vivo* using antibody-conjugated microbubbles. This technology, therefore, is also called ultrasound molecular imaging.

The safety of ultrasound molecular imaging using MBs and ultrasound cavitation treatment was investigated in a healthy porcine model. With confirmation of its safety using a clinical-grade ultrasound scanner and contrast agent, ultrasound cavitation treatment could be easily translated into clinical trials to improve diagnosis and chemotherapy delivery (4). In recent years, many preclinical studies, including diagnosis and drug delivery, especially cancer therapy, have been developed and are ongoing (5–7). Targeted CEUS promotes molecular medicine development. Pancreatic ductal adenocarcinoma (PDAC) is often associated with a poor prognosis due to its silent onset, and the diagnosis relies on anatomical changes, with difficulty discriminating between benign and malignant conditions. Bam et al. engineered a clinically translatable Thy1-targeted microbubble, as Thy1 has been previously validated as a clinically relevant biomarker for ultrasound imaging of PDAC. It specifically recognizes the thymocyte differentiation antigen (Thy1/CD90) overexpressed on the surface of vascular endothelial cells in PDAC tissues compared to normal and inflamed tissues (8).

In organ transplantation, although biopsy is still the gold standard for rejection diagnosis, its invasive nature prevents it from being a regular and repeatable method in the clinic. Targeted ultrasound imaging provides a convenient tool for physicians to determine important target expression in the allograft with a global view. Furthermore, quantitative information can be acquired from targeted ultrasound imaging rather than semiquantitative results from histochemical staining. Liao et al. designed a C4d-targeted microbubble and detected C4d deposition *in vivo* in a rat model of antibody-mediated rejection by CEUS imaging (9). For T-cell-mediated rejection, Grabner et al. combined anti-CD3 antibodies with MBs. CD3-targeted microbubbles allowed the detection of acute rejection as early as POD 2 in a rat renal transplantation model (10). However, using CD3 as the target identified any kind of CD4 and CD8 T cell, for example, cytotoxic T cells or regulatory T cells. In our study, we designed MBs coupled with anti-GzB antibodies, which were better able to reveal the terminal cytotoxic effect executed by the immune system. Using a murine heart transplant model, we assessed GzB distribution and quantified its expression at different time points post-transplantation.

## 2 Materials and methods

### 2.1 Preparation of MBs with or without anti-GzB antibodies

Commercially available streptavidin-coated MBs (MicroMarker Target Ready) were purchased from VisualSonics (Toronto, Canada). The anti-GzB antibody (Cat# BAF1865, R&D Systems, Minneapolis, MN) was biotinylated to allow conjugation with streptavidin MBs before use. Goat anti-mouse IgG antibody (ab150113, Abcam, Cambridge, UK) was used as an isotype control. Two types of MBs (MB_GzB_ and MB_IgG_) were prepared according to the manufacturer’s instructions. Briefly, 1 ml of saline was injected into the MicroMarker Target Ready vial, which was gently agitated for 40 s and left at room temperature for 5 min. Subsequently, 50 µg of antibodies were diluted into 1 ml. Next, 400 µl of activated MBs were mixed with 80 µl of diluted antibodies, followed by vortexing for 15 min at 4 °C. Unconjugated ligand was removed by centrifugal washing, after which 50 µl of dissolved MBs labeled with antibodies were used per animal in cardiac transplant recipients.

### 2.2 Animals and heart transplantation model

Male C3H and C57BL/6J mice were purchased from Gempharmatech Co., Ltd., Shanghai, China. All mice were maintained under standard conditions and fed rodent food and water, following the guidelines of the Animal Use and Care Committee of the Department of Laboratory Animal Science, Fudan University, Shanghai, China. Hearts were transplanted from C57BL/6J (allogeneic) or C3H (syngeneic) donors to C3H recipients. Intra-abdominal vascularized heterotopic mouse cardiac transplantation was performed as described previously (11, 12). Cardiac graft survival was determined by palpating the recipient’s abdomen daily. All animal experiments were approved by this committee and performed according to its recommendations.

### 2.3 Image acquisition

Ultrasound equipment and parameters: A TOSHIBA Aplio-500 Color Doppler ultrasound diagnostic instrument (Toshiba, Kawasaki-shi, Kanagawa, Japan) was employed using an L14-5 ultrawideband linear array probe (frequency, 5–14 MHz). The mechanical index (MI) of contrast-enhanced ultrasound ranged from 0.05 to 0.09, and all parameter settings were consistent during the inspection process: frequency, 12 MHz; grayscale MI, 0.06; frame rate, 8 fps; grayscale gain, 80; dynamic range (DR), 45; contrast MI, 0.08; frame rate, 8 fps; harmonic frequency, 5.5 MHz; contrast gain, 66; DR, 85.

Routine ultrasonography: Mice were anesthetized with 1% sodium pentobarbital (50 mg/ml) intraperitoneally before ultrasonography. Grayscale ultrasonography and color Doppler ultrasonography of all cardiac allografts were performed, and the sonographic manifestations (size, echo, blood flow distribution, etc.) were recorded.

Contrast-enhanced ultrasonography imaging: The probe was fixed on the largest four-chamber view of the cardiac allograft, and 50 μl of contrast agent was injected through the tail vein. The contrast-enhanced ultrasound function and timer were activated at the same time to record the contrast images. Since the echo intensity of the directly acquired sonogram was the sum of the signals of the circulating microbubbles and the target-bound microbubbles, ultrasound-induced microbubble destruction and replenishment were required to obtain the signal intensity of the target-bound microbubbles. Three minutes after the injection of the contrast agent, the dynamic image of the contrast medium was assessed for 10 s. Currently, the signal intensity of the obtained image was the sum of the number of free circulating microbubbles and the number of target-bound microbubbles (P1). Then, the flash function was activated to destroy the microbubbles in the cardiac imaging area with high-frequency ultrasound, and the contrast agent was allowed to fill the cardiac allograft imaging area again for 5 s after flashing. Then, the next 10 s dynamic sonogram was collected to obtain the image signal intensity representing free circulating microbubbles. The differential tissue enhancement (dTE; dTE = P1 − P2) was the amount of target-binding microvesicles. The images 10 s before and after flashing at 3 min and 6 min were collected for later analysis. All images were stored in standard DICOM format for subsequent offline quantitative analysis.

Quantitative analysis of images: Image analysis was performed using quantitative analysis software (version 2016) bundled with the Toshiba Aplio-500 instrument. In cases of overall enhancement of the cardiac allograft, the entire heart was selected as the region of interest (ROI). After the ROI was selected, the software automatically drew the corresponding dynamic enhancement time-intensity curve (TIC) and obtained quantitative parameters of time and intensity. The 10 s images before and after flashing at 3 min and 6 min were analyzed, and the peak intensity (PI) 1, PI2, valley intensity (VI) 1, and VI2 of the images at 3 min and 6 min were obtained. After selecting the ROI, the software automatically drew the corresponding dynamic enhancement TIC, obtained the time and intensity quantitative parameters, and calculated the intensity difference (dTE) before and after flashing, which was used for intergroup comparisons for different cardiac allograft groups.

Microbubbles: USphereTM Labeler was used to modify the biotin molecules or soften (avidin) the shell of Resonar, allowing antibody binding to give the microbubble specific adsorption ability.

**Figure 1:**
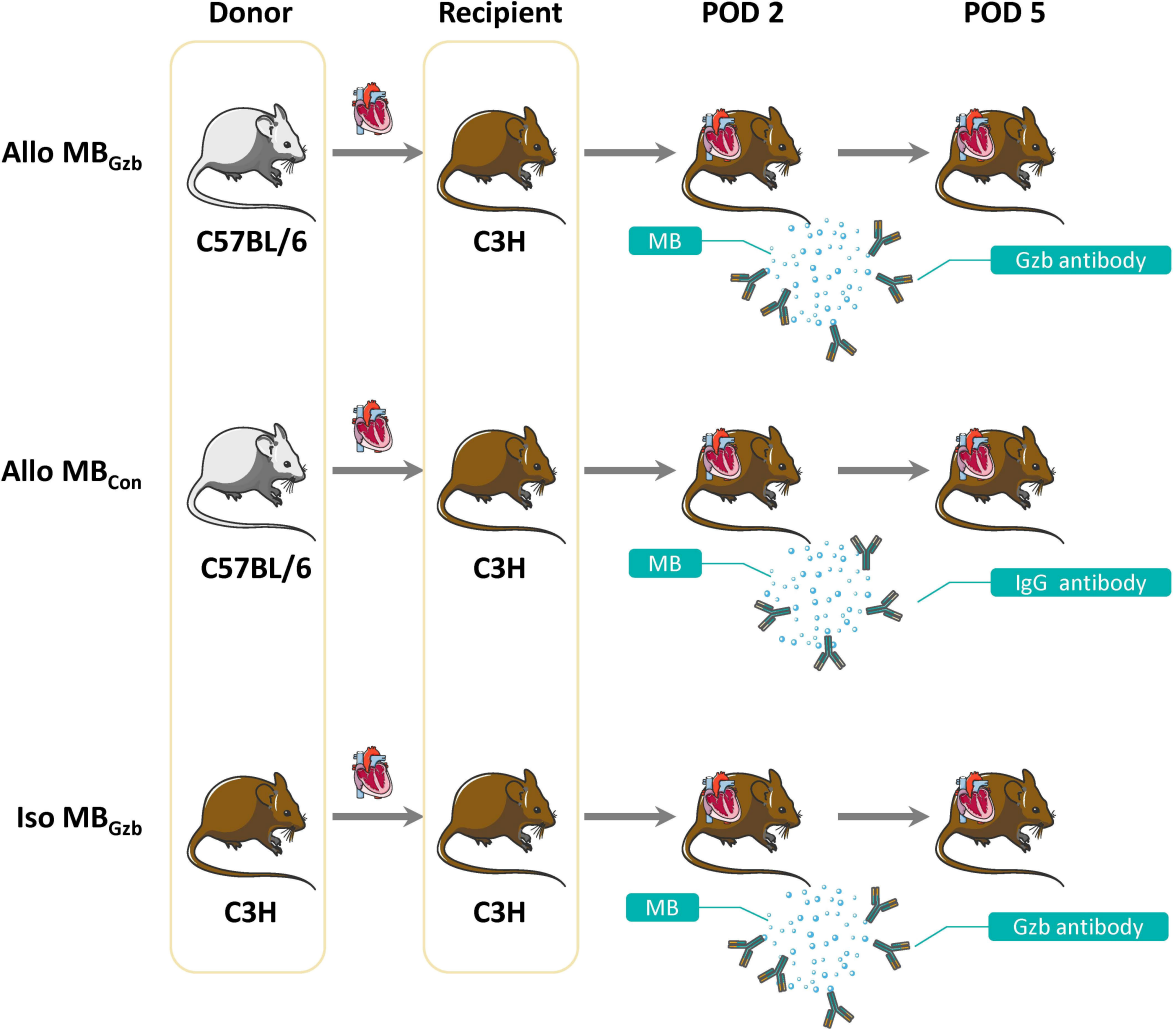
Study design. A total of 30 pairs of mice were divided into three groups: the Allo MB_Gzb_, Allo MB_con_, and Iso MB_Gzb_ groups (n = 10). All recipients underwent ultrasound examinations at PODs 2 and 5.

The groups and procedures are illustrated in **Figure 1**.

### 2.4 Repeatability of CEUS quantification

Twenty mouse cardiac grafts were subjected to two CEUS examinations and analyzed separately to assess the repeatability of CEUS. Interobserver agreement was expressed as the ICC, with ICC values ranging from +1 (100% agreement) to −1 (100% disagreement).

### 2.5 Histologic examination

All cardiac grafts were harvested on Days 2 and 5 post-transplant. Grafts were perfused for fixation and embedded in paraffin before being stained for GzB, myeloperoxidase (MPO), and CD8. Histologic changes and immunostaining in allograft tissue were examined by light microscopy. Quantification was made on cells/40× field.

### 2.6 Western blotting

Twenty micrograms of protein from heart homogenate were separated on 15% (wt/vol) polyacrylamide denaturing gels and electroblotted onto polyvinylidene fluoride membranes. The semiquantitative analysis (AlphaView Software 3.3, Cell Biosciences, Santa Clara, CA) results were expressed as the optical volume density (OD × mm^2^) normalized to β-actin (1:5,000, Servicebio, Wuhan, China). The antibodies used were goat anti-granzyme B (1:500, BAF1865, R&D, Minneapolis, MN) and rabbit anti-IL-6 (1:1,000, ab229381, Abcam, Cambridge, UK). The details are described in our previous study (13).

### 2.7 Statistical analysis

The results are expressed as the mean ± standard deviation. Normality tests were carried out, and statistical analysis of the data was performed using one-way ANOVA and Dunnett’s test analysis of variance using Prism 9.0 software (GraphPad Software, San Diego, CA). P <0.05 was considered statistically significant.

## 3 Results

### 3.1 Repeatability of CEUS quantification

According to the Bland-Altman method, there was good interobserver agreement for the PI and VI values, with ICCs of 0.99 and 0.98, respectively (**Table 1**).

**Table 1:** Interobserver agreement of quantitative CEUS parameters.

	ICC	95% Confidence interval	*p* value
PI	0.999	0.999–1.000	<0.001
VI	0.985	0.964–0.994	<0.001

ICC, intraclass correlation coefficient; PI, peak intensity; VI, valley intensity.

### 3.2 CEUS molecular imaging

**Figure 2:**
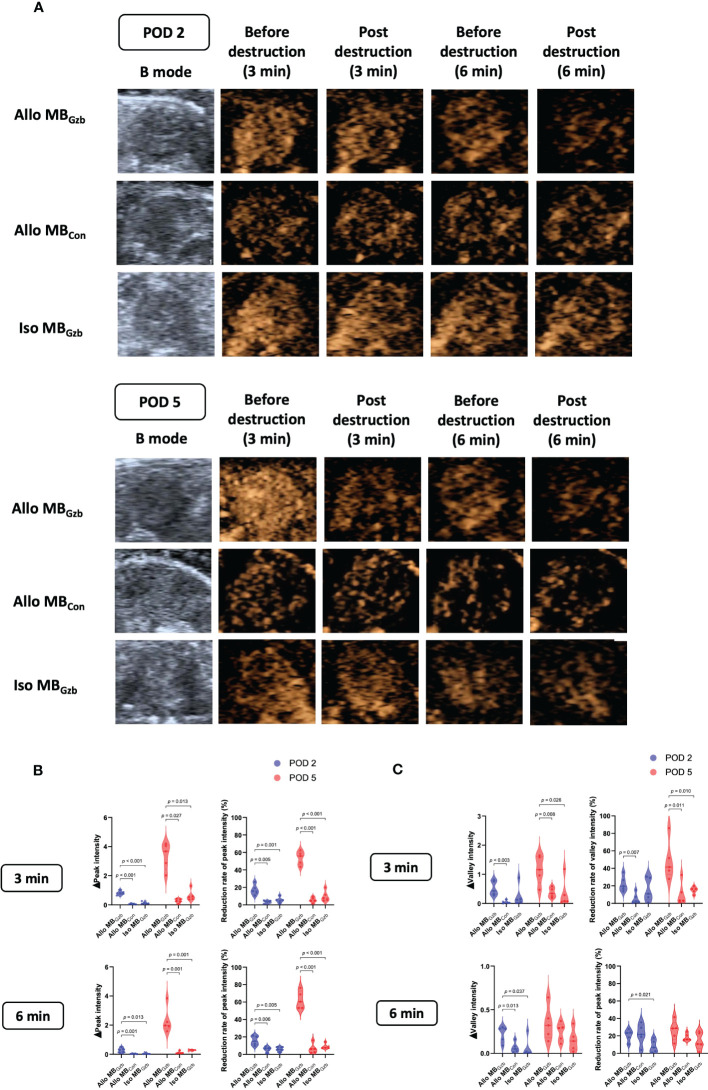
Molecular imaging of CEUS and quantitative analysis. B-mode ultrasound and CEUS were performed at PODs 2 and 5. After rapid injection of MBs with anti-granzyme B antibody or isotype control antibody *via* the tail vein, molecular signals were obtained by CEUS before and after a flash pulse **(A)**. The decreases in peak **(B)** and valley intensities **(C)** are illustrated.

A total of 32 mice were successfully subjected to an ultrasound examination. Two mice were excluded from the data analysis due to accidental deaths caused by anesthetic complications before the ultrasound examination. We first performed a B-mode Doppler ultrasound. The average maximal size of the cardiac section was 30.20 ± 0.25 mm^2^. Next, we collected contrast imaging data. The myocardium showed rapid enhancement without localized perfusion abnormalities after a bolus injection of MBs that reached maximum in a short period of time. Then, the myocardium showed a slow decay in intensity for both MB_Gzb_ and MB_con_. After MB injection, we observed and collected data at 3 and 6 min before and after the flash pulse. In the allogeneic MB_Gzb_ group, a significant decrease in the contrast signal in the myocardium was observed, followed by a slow reflow of MBs a few seconds after the flash pulse (**Figure 2A**). Given that antibodies bind to antigens, MB_Gzb_ should stay in the local myocardium, where granzyme B exists. The binding of MB_Gzb_ dramatically increased after the flash pulse. Although circulating MBs reflowed into the transplanted heart, no further MB_Gzb_ bound to granzyme B in those few seconds. Therefore, we calculated the decreased MB intensity in the myocardium to reflect the quantity of bound MBs. Quantitative analysis revealed that the reduction in peak intensity was significantly higher in the allogeneic MB_Gzb_ group than in the allogeneic MB_con_ group and isogeneic MB_con_ group in terms of number and percentage at PODs 2 and 5 (**Figure 2B**). In addition, we analyzed the valley intensity data. Although the trend was similar to that of the peak intensity, the significance was lower (**Figure 2C**), suggesting that the peak intensity was a better parameter than the valley intensity.

### 3.3 Histology and immunohistology staining in cardiac grafts

**Figure 3:**
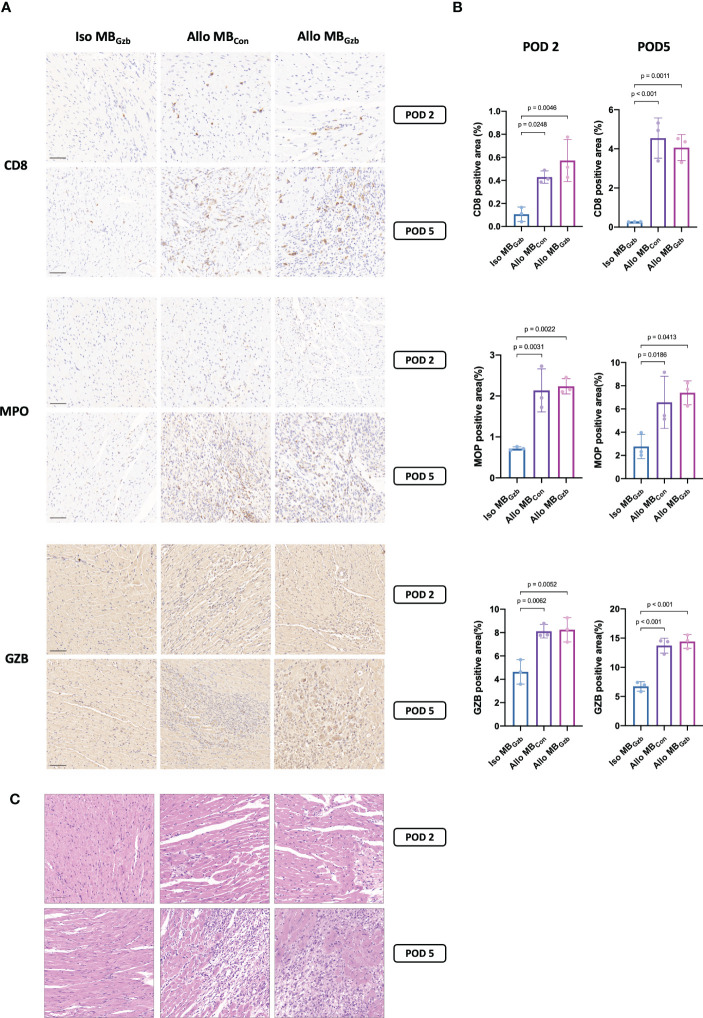
CD8, MPO, and GzB staining in cardiac grafts. CD8, MPO, and GzB immunohistochemistry **(A)** and H&E **(C)** staining and quantitative analysis **(B)** of cardiac grafts in the Iso MB_Gzb_, Allo MB_Gzb_ and Allo MB_con_ groups at POD 2 and POD 5. Scale bar = 50 μm. MPO, myeloperoxidase; GZB, granzyme B.

To characterize the histologic features and intensity of rejection, CD8, MPO, and granzyme B in each group were stained. Weak positivity for CD8 was observed in the isogeneic MB_con_ group at PODs 2 and 5, while in the allogeneic MB_con_ and MB_Gzb_ groups, stronger positivity for CD8 was observed. There were also more infiltrating cytotoxic CD8^+^ T cells in allografts than in isografts. In addition, more cytotoxic CD8^+^ T cells infiltrated myocardial tissues at POD 5 than at POD 2 in the allogeneic groups, suggesting worsening TCMR after heart transplantation. There was no significant difference between the allogeneic MB_con_ and MB_Gzb_ groups. MPO is a critical inflammatory enzyme that triggers both oxidative stress and inflammation in the process of ischemic reperfusion injury and rejection after heart transplantation and is present in neutrophils (13). MPO staining showed more infiltrated neutrophils in the allogeneic groups than in the isogeneic groups and more severe inflammation on Day 5 than on Day 2, which was consistent with the intensity of graft injuries observed through CD8 staining. Moreover, the deposition of granzyme B was also observed to be highly positive in the allogeneic MB_con_ and MB_Gzb_ groups. At POD 2, granzyme B was focally deposited, while at POD 5, it was obviously increased and showed diffuse staining. In the isogeneic MB_Gzb_ group, little granzyme B staining was observed due to the slight rejection after transplantation and the low amount of cytotoxic T-cell infiltration (**Figure 3A**). Quantitative analysis more intuitively showed this comparison (**Figure 3B**). We also showed the tissue injuries and immune cell infiltration caused by acute rejection with H&E staining (**Figure 3C**).

### 3.4 Cytokine expression in cardiac grafts

**Figure 4:**
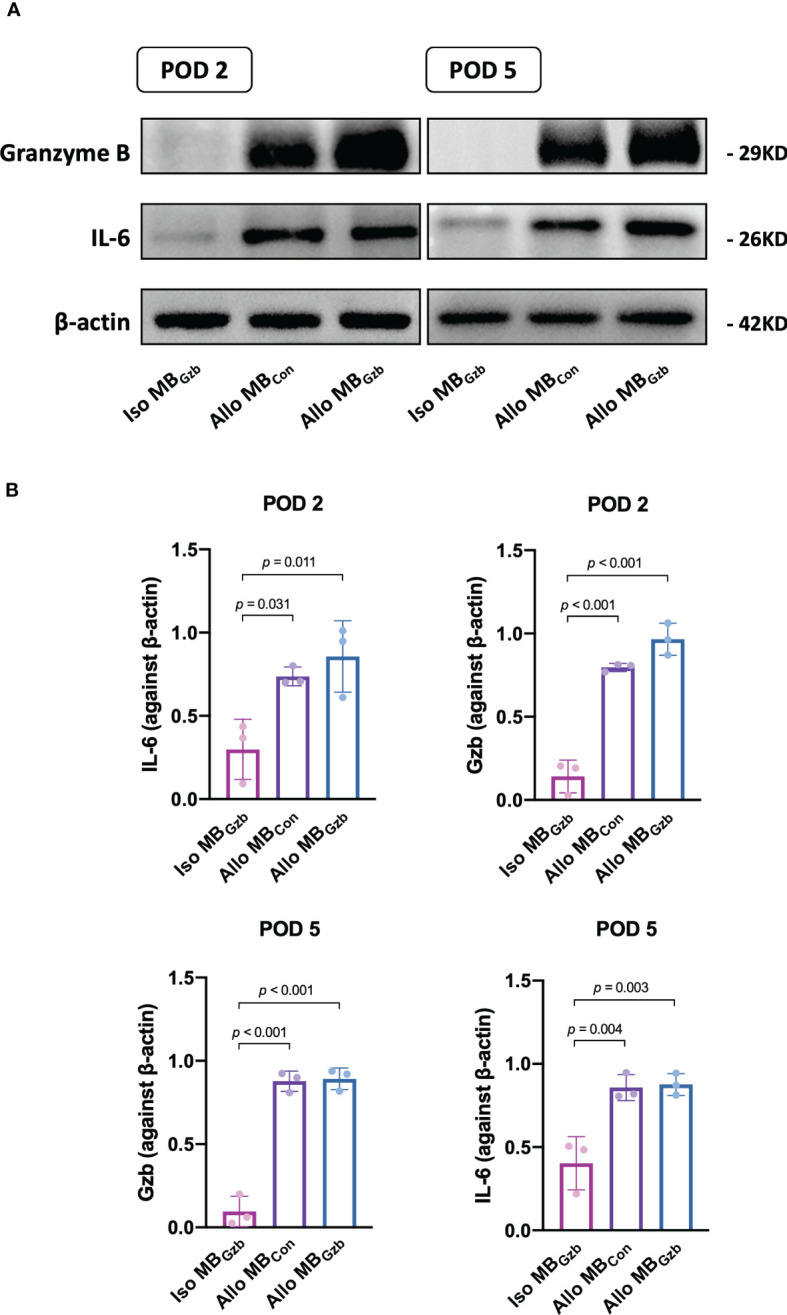
GzB and IL-6 protein expression in cardiac grafts. Western blot **(A)** analysis of granzyme B and IL-6 in the Iso MB_Gzb_, Allo MB_Gzb_ and Allo MB_con_ groups at PODs 2 and 5 **(B)**.

We further explored the molecular changes after heart transplantation and tested the expression of granzyme B and the cytokine IL-6 (**Figure 4A**). At POD 2, the expression of granzyme B and IL-6 was upregulated in the allogeneic MB_con_ and MB_Gzb_ groups. In the isogeneic MB_Gzb_ group, hardly any granzyme B or IL-6 was expressed compared to the allogeneic groups. The expression of cytokines in allografts at POD 5 was similar to that at POD 2 in both qualitative and quantitative analyses (**Figure 4B**).

## 4 Discussion

In this study, we developed a noninvasive method using targeted CEUS imaging *in vivo* to quantitatively assess granzyme B expression in cardiac grafts. We injected MBs labeled with granzyme B antibodies, detected them at 3 and 6 min, and quantified the peak and valley intensities of MBs in the myocardium before and after the flash pulse. In terms of the acquisition time and method of contrast-enhanced imaging, our experience in mouse models suggests that a time frame of 3 min after injection of targeted ultrasound microbubbles is long enough for satisfactory binding with the target protein and reaching a stable plateau phase in terms of ultrasound microbubble signal intensity. Therefore, we chose to collect images at 3 and 6 min after the contrast agent injection. Second, we considered the perfusion dynamics of transplanted hearts in mice, the kinetics of microbubbles, nonspecific binding, and the influence of the flash pulse on microbubbles within the circulation, and we indirectly reflected the number of targeted microbubbles by collecting and calculating the difference in microbubble intensity before and after the flash pulse in the heart. The image acquisition interval between P1 and P2 was set to 10 s, which ensured that microbubbles within the circulation were refilled and evenly distributed throughout the circulation system, including the transplanted heart, after the flash pulse and had reached a stable plateau phase in terms of ultrasound microbubble signal intensity. However, stable targeting of microbubbles requires a longer time (approximately 3 min). Moreover, we selected the transverse plane for image acquisition, which included the transplanted heart and the transverse section of the abdominal blood vessels, compared to the longitudinal section of the abdomen. This approach minimized the damage caused by the flash pulse to microbubbles within the systemic circulation outside the transplanted heart while ensuring the destruction of all microbubbles within the target heart area. Through these methods, we minimized the impact of various factors on the results and ensured that the difference between P1 and P2 reflected the true number of targeted microbubbles as much as possible.

Granzyme B (GzB), a serine protease released primarily by cytotoxic T cells as an important allograft-killing mechanism during rejection, may serve as a rejection biomarker. Perforin forms a pore in the target cell, allowing for the delivery of GzB. Granzyme B cleaves multiple apoptotic pathway components that converge on the cleavage of caspase 3, the executioner caspase (14). As early as the 20th century, Krams et al. found that the mRNA expression of GzB in biopsy specimens was significantly higher in the acute rejection group of liver allograft recipients (15). In addition to T-cell-mediated rejection, GzB also participates in antibody-mediated rejection. In a cohort of living donor first renal allograft recipients without a prior history of rejection, intragraft GzB^+^ cell count and GzB mRNA expression were significantly higher in the chronic antibody-mediated rejection group than in the stable graft function group (16). Therefore, decision-making requires the discovery and even quantitative detection of GzB expression in the allograft as early as possible. There is a granzyme B-responsive fluorescent probe for the noninvasive early diagnosis of transplant rejection. The probe can detect the activity of granzyme B in the myocardium of mice using fluorescence imaging. The probe shows high sensitivity and specificity for granzyme B and can monitor the progression of rejection over time (17). Chen et al. invented a responsive second near-infrared (NIR-II) fluorescent nanosensor (ErGZ) that allows ratiometric *in vivo* fluorescence sensing of granzyme B to detect early allograft rejection (18).

We found that the allogeneic MB_Gzb_ group showed a significant decrease in the contrast signal in the myocardium after the flash pulse, indicating the binding of MB_Gzb_ to granzyme B. We also found that the reduction in peak intensity was significantly higher in the allogeneic MB_Gzb_ group than in the allogeneic MB_con_ group and the isogeneic MB_con_ group at PODs 2 and 5, and the peak intensity was a better parameter than the valley intensity for quantifying the binding MBs as it had higher significance and less variability. When the instrument collects microbubble intensity signals, the recorded image is a dynamic image of a 10-second period. Based on the perfusion of transplanted hearts in mice and the movement of microbubbles within the circulation, some microbubbles may be occluded by the microbubbles in front of them, resulting in signal intensity lower than the actual number of microbubbles. Therefore, the trough value reflects the state with the most severe loss of microbubble signals, while the peak value is the best parameter to truly reflect the number of microbubbles.

In previous studies, our group performed a series of noninvasive studies using CEUS for transplant allograft detection in the clinic. We established some mathematical models to assess allograft immune status qualitatively and quantitatively in kidney transplant patients in terms of acute and chronic rejections (19, 20). Therefore, we wanted to further investigate dynamic molecular imaging in the rejection process. During acute cellular rejection, graft damage is mediated by recipient cytotoxic CD8^+^ T cells that are activated by alloantigens and target allogeneic cells for killing. Acute cellular rejection episodes may occur at any time during the allograft lifespan, even years after immunological quiescence (21). Acute cellular rejection can be effectively treated with anti-rejection medicines targeting T cells (e.g., steroids, thyroglobulin). Therefore, it is of great importance to detect the level of anti-allograft T-cell responses at an early stage of acute cellular rejection. The mechanism by which activated cytotoxic T cells engage and kill target cells is well studied and involves the release of cytolytic granules containing perforin. Previous studies revealed that the level of GzB^+^ lymphocytes in allografts was significantly higher in stages IA and IB than in control biopsies and predicted rapid progression to severe acute cellular rejection (TCMR grade II or higher) (22, 23). Therefore, it is valuable to obtain dynamic and repeatable quantitative data on GzB expression *in vivo* using noninvasive methods such as targeted ultrasound imaging.

However, our method still has some limitations. Although we tried to minimize the influence of the destruction of microbubbles, the data cannot be perfectly exact. More accurate measurements need to be explored in the future.

In conclusion, we report a noninvasive, comprehensive, and quantitative method for determining GzB expression in cardiac allografts through targeted ultrasound imaging. This method might facilitate the diagnosis of allograft rejection in the future.

## Data availability statement

The original contributions presented in the study are included in the article/Supplementary Material. Further inquiries can be directed to the corresponding authors.

## Ethics statement

The animal study was reviewed and approved by Provisos or Reservations of Animal Ethics Committee, Zhongshan Hospital, Fudan University.

## Author contributions

Ultrasound examination and analysis, YJ. Animal model, JH and PG. Biological and molecular experiments, LL and PG. Writing-original draft preparation, YJ, LL, and CY. Writing-review and editing, JH. Study design, CY, JH, and LJ. Funding, CY. All authors contributed to the article and approved the submitted version.
